# CXCL1 Promotes Osteoblast Autophagy and Inhibits Ferroptosis Through the Activation of the TGF‐β/Smad Signalling Pathway

**DOI:** 10.1111/jcmm.70883

**Published:** 2025-10-30

**Authors:** Zhiqiang Zhou, Yi Wang, Zhiqi Gao, Qiong Chen, Han Wu, Jiao Sun, Huilan Li, Dong Liu, Yixin Shen

**Affiliations:** ^1^ Department of Orthopedics The Second Affiliated Hospital of Soochow University Suzhou China; ^2^ Department of Radiology, Suzhou Research Center of Medical School, Suzhou Hospital, Affiliated Hospital of Medical School Nanjing University Suzhou China; ^3^ Department of Anesthesiology The Second Affiliated Hospital of Soochow University Suzhou China; ^4^ Department of Orthopedics The Fourth Affiliated Hospital of Soochow University Suzhou China; ^5^ Department of Orthopedics Suzhou Xiangcheng People's Hospital Suzhou China; ^6^ Suzhou High‐Tech Zone Yangshan Community Health Service Center Suzhou China

**Keywords:** autophagy, CXCL1, ferroptosis, osteoblast, TGF‐β/Smad pathway

## Abstract

Osteoblast dysfunction plays a central role in osteoporosis. CXC chemokine ligand 1 (CXCL1), a known inflammatory mediator, is increasingly recognised for its role in bone homeostasis. However, its influence on osteoblast survival mechanisms such as ferroptosis and autophagy remains unclear. This study explores the role of CXCL1 in promoting osteoblast differentiation and activity by inhibiting ferroptosis and enhancing autophagy via the TGF‐β/Smad signalling pathway. Primary rat osteoblasts were treated with recombinant CXCL1, shRNA constructs and pathway modulators, such as Galunisertib, Fer‐1 and Chloroquine (CQ). Osteoblast differentiation, autophagy, ferroptosis and TGF‐β/Smad pathway activity were evaluated using qPCR, western blotting, staining and densitometric analysis. CXCL1 knockdown impaired osteoblast proliferation and differentiation, while increasing intracellular iron and ROS and enhancing ACSL4 expression, indicative of ferroptosis. These effects were partially reversed by Fer‐1. Besides, CXCL1 activated the TGF‐β/Smad signalling cascade, and Galunisertib inhibited this signalling and partially suppressed CXCL1‐induced effects. Furthermore, CXCL1 promoted autophagy via increased Beclin‐1 and LC3B and reduced p62, which mitigated ferroptosis and supported osteogenesis. Our findings suggest that CXCL1 promotes osteoblast differentiation by inhibiting ferroptosis and enhancing autophagy through activation of the TGF‐β/Smad signalling pathway. Collectively, our results highlight CXCL1 as a promising therapeutic target for osteoporosis.

## 1 Introduction

Osteoporotic fractures, commonly referred to as fragility fractures, are caused by minimal trauma and often indicate advanced osteoporosis. These fractures reflect a localised bone pathology that arises due to systemic reductions in bone density and strength [1, 2, 3]. Osteoporosis is primarily driven by decreased osteoblast function and overactive osteoclast activity, disrupting the balance between bone formation and resorption [4]. Recent studies have highlighted the importance of exploring osteoblast cell death mechanisms, including ferroptosis, apoptosis and autophagy, to identify novel therapeutic targets for osteoporosis [5, 6, 7, 8].

Ferroptosis is an iron‐dependent, regulated form of cell death characterised by the accumulation of reactive oxygen species (ROS) and lipid peroxidation [9]. Unlike apoptosis, which features nuclear condensation and DNA fragmentation, ferroptosis disrupts cell membrane integrity through lipid damage [10, 11]. Research indicates that iron overload disturbs bone formation and promotes osteoclast‐mediated degradation, thereby exacerbating bone loss [12, 13]. Elevated intracellular iron levels are known to exacerbate oxidative stress, triggering ferroptosis and bone loss. Understanding the regulatory mechanisms of ferroptosis in osteoblasts is essential for the development of targeted interventions for osteoporosis.

CXC chemokine ligand 1 (CXCL1), also known as growth‐regulated oncogene‐α (Gro‐α), is a member of the CXC chemokine family [14]. CXCL1 is involved in diverse biological processes, including inflammation, angiogenesis and cell migration, and plays a prominent role in the development of inflammatory diseases and tumour progression [15]. Although research has extensively examined the role of CXCL1 in cancer, limited studies have explored its effects on bone metabolism and osteoblasts. Notably, CXCL1 activates the CXC chemokine receptor 2 (CXCR2), which has been linked to the maturation of osteoclasts, suggesting a potential role for CXCL1 in bone remodelling [16].

Given the known role of CXCL1 in cellular signalling and its emerging implications in bone health, investigating its potential regulation of osteoblast survival and differentiation is crucial. Preliminary evidence indicates that CXCL1 may influence key signalling pathways involved in osteoblast function, including the TGF‐β/Smad pathway, which plays a pivotal role in bone formation and cell survival. This study hypothesises that CXCL1 promotes osteoblast autophagy and suppresses ferroptosis via activation of the TGF‐β/Smad signalling pathway, thereby supporting osteoblast differentiation and survival. By elucidating the mechanisms through which CXCL1 regulates ferroptosis and autophagy, this research aims to identify a potential therapeutic strategy for osteoporosis.

## 2 Materials and Methods

### 2.1 Chemicals, Reagents, and Antibodies

The antibodies utilised in this study were procured from Abcam (Cambridge, UK), Cell Signalling Technology (Shanghai, China), and Proteintech (Chicago, USA). The Cell Counting Kit‐8 (CCK‐8) was obtained from Solarbio (Beijing, China). The fluorescent dye 2′,7′‐Dichlorodihydrofluorescein diacetate (DCFH‐DA) was purchased from Beyotime (Shanghai, China). Puromycin, polybrene, the Annexin V‐based flow cytometry (FACS) apoptosis detection kit, the caspase‐3 activity assay kit and all general cell culture reagents were acquired from Sigma‐Aldrich (St. Louis, MO, USA). TRIzol reagent, Lipofectamine 2000 and other transfection reagents were provided by Thermo Fisher Scientific and Invitrogen (Shanghai, China). All cell culture media and supplements were sourced from Gibco (Suzhou, China).

### 2.2 Primary Osteoblast Isolation and Cell Culture

Primary rat calvarial osteoblasts were purchased from Wuhan Punosai and cultured in DMEM supplemented with 10% fetal bovine serum (FBS), 100 U/mL penicillin and 100 μg/mL streptomycin at 37°C in a humidified atmosphere of 5% CO_2_. Cells were sub‐cultured at a 1:2 ratio when 80% confluent. Passage numbers 2–4 were used for experiments. For osteogenic differentiation, ascorbic acid (50 μg/mL) and β‐glycerophosphate (10 mM) were added to the medium.

### 2.3 Pharmacological Treatments

Cells were treated with the following reagents: recombinant rat CXCL1 (10 ng/mL) for 12 h; the TGF‐β receptor I kinase inhibitor Galunisertib (LY2157299, 10 μM) for 12 h; the ferroptosis inhibitor Ferrostatin‐1 (Fer‐1, 10 μM) for 12 h; and the autophagy inhibitor Chloroquine (CQ, 100 μM) for 12 h. All treatments were administered to cells at 70%–80% confluency. Vehicle‐treated cells (0.1% DMSO) served as controls. All treatments were performed in triplicate.

### 2.4 CXCL1 Knockdown Via shRNA

Three shRNA sequences targeting CXCL1 were synthesized by Sangon Biotechnology (Shanghai). Using the BR‐V108 lentiviral vector, obtained from Genechem (Shanghai), we generated shRNA‐expressing vectors and verified them via DNA sequencing. The lentiviral particles were subsequently produced and used to infect osteoblasts. After 48 h, puromycin (5.0 μg/mL) was added to select for stable cells for 8 days. Over 95% CXCL1 knockdown in stable cells was verified by western blotting and RT‐PCR analyses.

### 2.5 Cell Viability Assay

Osteoblast viability was assessed using the CCK‐8 assay (Solarbio, CA1210). Cells (2000/well) were seeded in 96‐well plates. After treatments as indicated in the results, cells were incubated with 10 μL of CCK‐8 reagent per well for 2 h at 37°C. Absorbance was then measured at 450 nm using a microplate reader.

### 2.6 Colony Formation Assay

Cells (400–1000/well) were plated in 6‐well plates (triplicate for each condition) and cultured for 14 days. Colonies were then fixed with 4% paraformaldehyde, stained with Giemsa, and counted under a microscope. A colony was defined as a cluster of > 50 cells.

### 2.7 Alkaline Phosphatase (ALP) and Alizarin Red Staining

ALP staining was performed per the manufacturer's protocol (MesGen Biotechnology, MAP1345). After fixation, cells were stained in the dark for 15–30 min and observed microscopically. For Alizarin Red staining, cells were fixed and incubated with 2% Alizarin Red S solution (pH 4.2) for 30 min at room temperature and then rinsed thoroughly with distilled water to remove non‐specific staining. For quantification, Alizarin Red S was dissolved in 10% cetylpyridinium chloride, and the absorbance was measured at 562 nm.

### 2.8 Western Blotting

Total proteins were extracted using RIPA buffer (Beyotime) with protease and phosphatase inhibitors. Equal amounts of protein (20–30 μg) were separated on 10% SDS‐PAGE gels and transferred to PVDF membranes (Millipore). Membranes were blocked with 5% non‐fat milk and incubated overnight at 4°C with the following primary antibodies: CXCL1 (Abcam, ab86436, 1:1000), GPX4 (Cell Signalling Technology, #52455, 1:1000), SLC7A11 (Abcam, ab175186, 1:1000), ACSL4 (Abcam, ab155282, 1:1000), TGFβ1 (Abcam, ab92486, 1:1000), BMP2 (Abcam, ab14933, 1:1000), Smad2/3 and p‐Smad2 (Ser465/467), p‐Smad3 (Ser423/425) (Cell Signalling Technology, #3108, #8828, 1:1000), Beclin‐1 (Cell Signalling Technology, #3495, 1:1000), LC3B (Cell Signalling Technology, #3868, 1:1000), p62/SQSTM1 (Abcam, ab56416, 1:1000), β‐actin (Proteintech, 66,009–1‐Ig, 1:5000) as a loading control. Secondary HRP‐conjugated antibodies were used at 1:5000, and bands were visualised using ECL substrate (Thermo). Densitometric quantification of Western blot bands was performed using ImageJ software.

### 2.9 Quantitative Real‐Time PCR (qPCR)

Total RNA was isolated using TRIzol (Invitrogen) and reverse transcribed into cDNA using the HiScript II RT SuperMix (Vazyme). qPCR was performed with ChamQ SYBR qPCR Master Mix (Vazyme) on a QuantStudio 5 system (Applied Biosystems). The thermocycling conditions were as follows: initial denaturation at 95°C for 30 s, followed by 40 cycles of 95°C for 10 s and 60°C for 30 s. A melt curve analysis was performed to confirm amplification specificity. Primer sequences are listed in Table S1. GAPDH served as the internal control. Relative expression levels were calculated using the 2^−ΔΔCt^ method.

### 2.10 Iron and ROS Measurements

Intracellular Fe^2+^ levels were measured using the Iron Assay Kit (Abcam, ab83366) according to the manufacturer's instructions. Briefly, cells were lysed, and the lysate was incubated with an iron probe. The absorbance was measured at 593 nm. Reactive oxygen species (ROS) were detected using the DCFH‐DA probe (Beyotime, S0033M). Cells were incubated with 10 μM DCFH‐DA for 20 min at 37°C and then washed with serum‐free medium. Fluorescence was measured by flow cytometry (BD FACSCalibur) or observed under a fluorescence microscope (Leica DMi8).

### 2.11 Immunofluorescence

Osteoblasts were seeded on sterile glass coverslips in 24‐well plates and treated according to experimental conditions. After treatment, cells were fixed with 4% paraformaldehyde for 15 min at room temperature, permeabilized with 0.1% Triton X‐100 for 10 min, and blocked with 5% BSA for 1 h. Cells were incubated overnight at 4°C with primary antibodies against LC3B (CST, #3868, 1:200) and GPX4 (CST, #52455, 1:200). After washing, Alexa Fluor‐conjugated secondary antibodies (Invitrogen, 1:500) were applied for 1 h at room temperature in the dark. Nuclei were counterstained with DAPI (Beyotime) for 5 min. Images were captured using a fluorescence microscope (Leica DMi8) and analyzed with ImageJ.

### 2.12 Statistical Analysis

Experiments were repeated at least three times. Data are expressed as mean ± SD. Statistical significance was determined using one‐way ANOVA followed by Tukey's multiple comparisons test in GraphPad Prism software (version 9.0). A *p*‐value < 0.05 was considered significant.

## 3 Results

### 3.1 CXCL1 Enhances Osteoblast Proliferation and Differentiation

**FIGURE 1:**
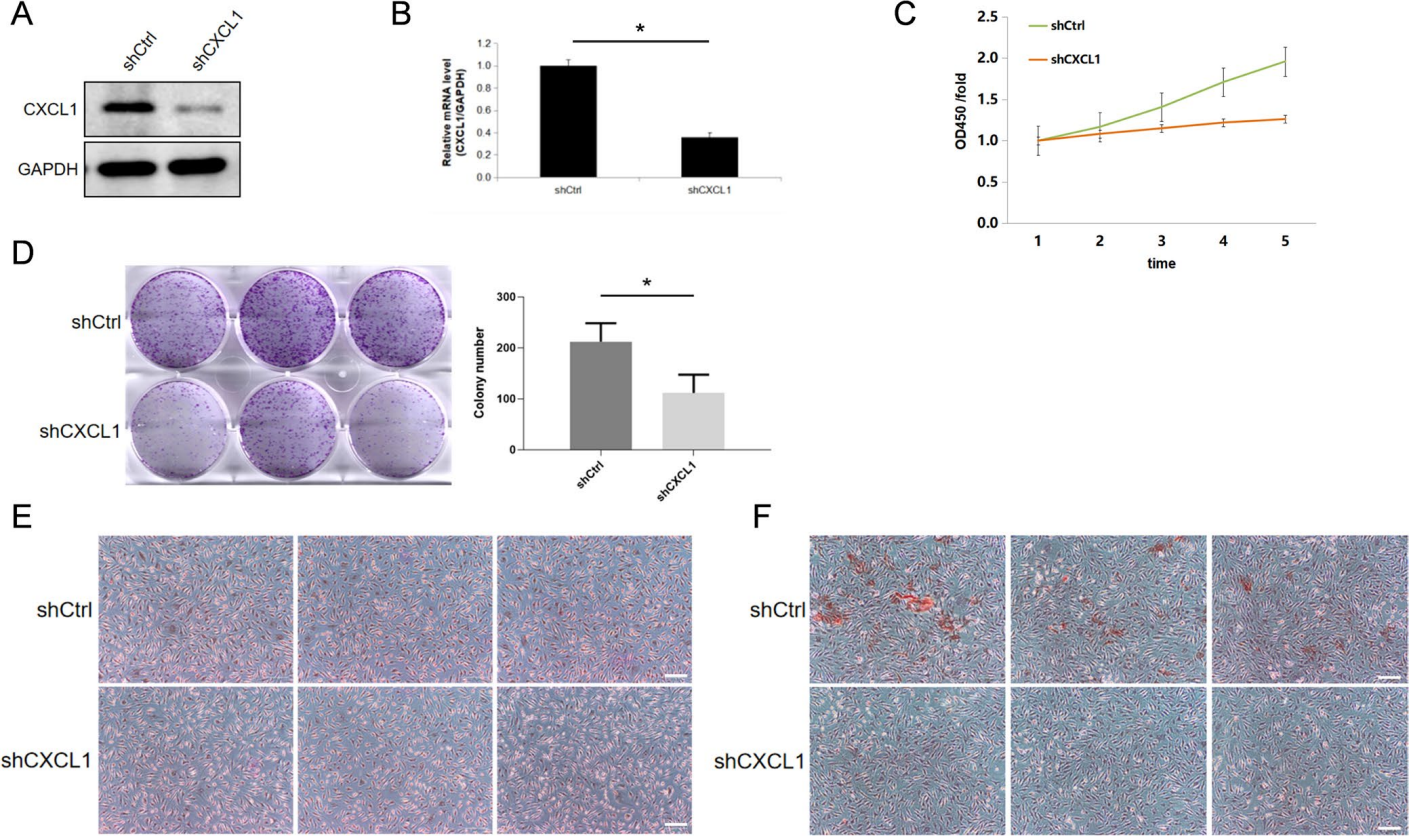
CXCL1 promotes osteoblast proliferation and differentiation. Osteoblasts were divided into the control group (shCtrl) and the CXCL1 knockdown group (shCXCL1) using lentiviral plasmid infection. Western blot analysis (A, B) was conducted to detect CXCL1 protein expression, and qPCR was used to assess CXCL1 mRNA levels. The CCK‐8 assay (C) was performed to evaluate osteoblast proliferation activity. Colony formation assays (D) were conducted to assess the number of osteoblasts. ALP staining and Alizarin Red staining (E, F) were used to evaluate the differentiation capacity of osteoblasts. Expressions of the listed proteins were quantified and normalised to the loading control. Quantified values were mean ± standard deviation (SD, *n* = 5). Statistical significance was indicated by *p < 0.05 compared to the “shCtrl” group. These experiments were repeated five times, consistently yielding similar results. Scale Bar = 200 μm.

To investigate CXCL1's role, we performed knockdown experiments in osteoblasts using lentiviral infection and confirmed reduced CXCL1 expression at both mRNA and protein levels (Figure 1A,B). CCK‐8 assays showed a significant reduction in osteoblast proliferation over 5 days post‐CXCL1 knockdown (Figure 1C), whereas colony formation assays demonstrated a decrease in osteoblast proliferation (Figure 1D). ALP and Alizarin Red staining showed significantly decreased differentiation, as evidenced by reduced ALP activity and fewer calcified nodules (Figure 1E,F). Together, these findings indicate that CXCL1 supports osteoblast proliferation and differentiation.

### 3.2 CXCL1 Inhibits Ferroptosis in Osteoblasts to Promote Differentiation

**FIGURE 2:**
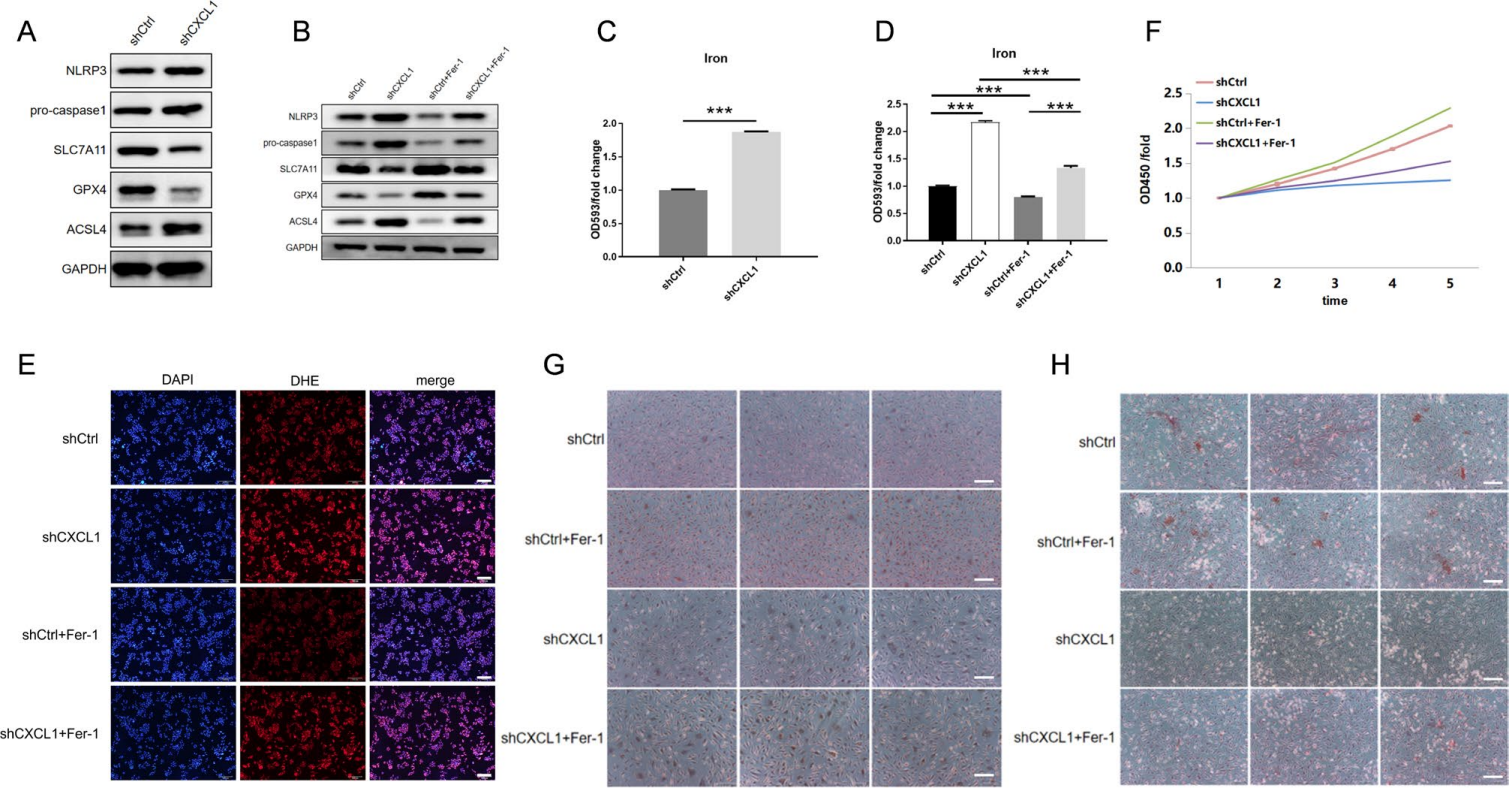
CXCL1 promotes osteoblast differentiation by inhibiting ferroptosis. Osteoblasts were divided into the following groups: Control (shCtrl), CXCL1 knockdown (shCXCL1), ferroptosis inhibitor treatment (shCtrl+Fer‐1), and CXCL1 knockdown with ferroptosis inhibitor treatment (shCXCL1+Fer‐1). Western blot (A, B) was used to detect the expression of ACSL4, SCL7A11, and GPX4 proteins. Intracellular iron content changes were measured using an iron ion assay kit (C, D). Oxidative stress levels were assessed using an ROS assay kit (E). The CCK‐8 assay (F) was performed to evaluate osteoblast proliferation. ALP and Alizarin Red staining (G, H) were used to determine the differentiation capacity of osteoblasts. Quantified values were mean ± standard deviation (SD, *n* = 5). Statistical significance was indicated by ***p < 0.01 compared to the “shCtrl” group. These experiments were repeated five times, consistently yielding similar results. Scale Bar = 200 μm.

Next, we examined CXCL1's influence on ferroptosis, focusing on ferroptosis‐related markers ACSL4, SCL7A11 and GPX4. CXCL1 knockdown increased ACSL4 expression while decreasing SCL7A11 and GPX4 levels (Figure 2A). Furthermore, CXCL1 deficiency elevated intracellular iron (Figure 2C). Treating cells with the ferroptosis inhibitor Fer‐1 (10 μM, 12 h) partially reversed these effects, restoring ferroptosis marker levels and attenuating iron increase (Figure 2B,D). Moreover, Fer‐1 treatment significantly reduced ROS accumulation (Figure 2E) and mitigated the proliferation and differentiation deficits caused by CXCL1 knockdown (Figure 2F–H). These findings suggest that CXCL1 inhibits ferroptosis to promote osteoblast function.

### 3.3 CXCL1 Activates the TGF‐β/Smad Pathway to Inhibit Ferroptosis and Enhance Differentiation

**FIGURE 3:**
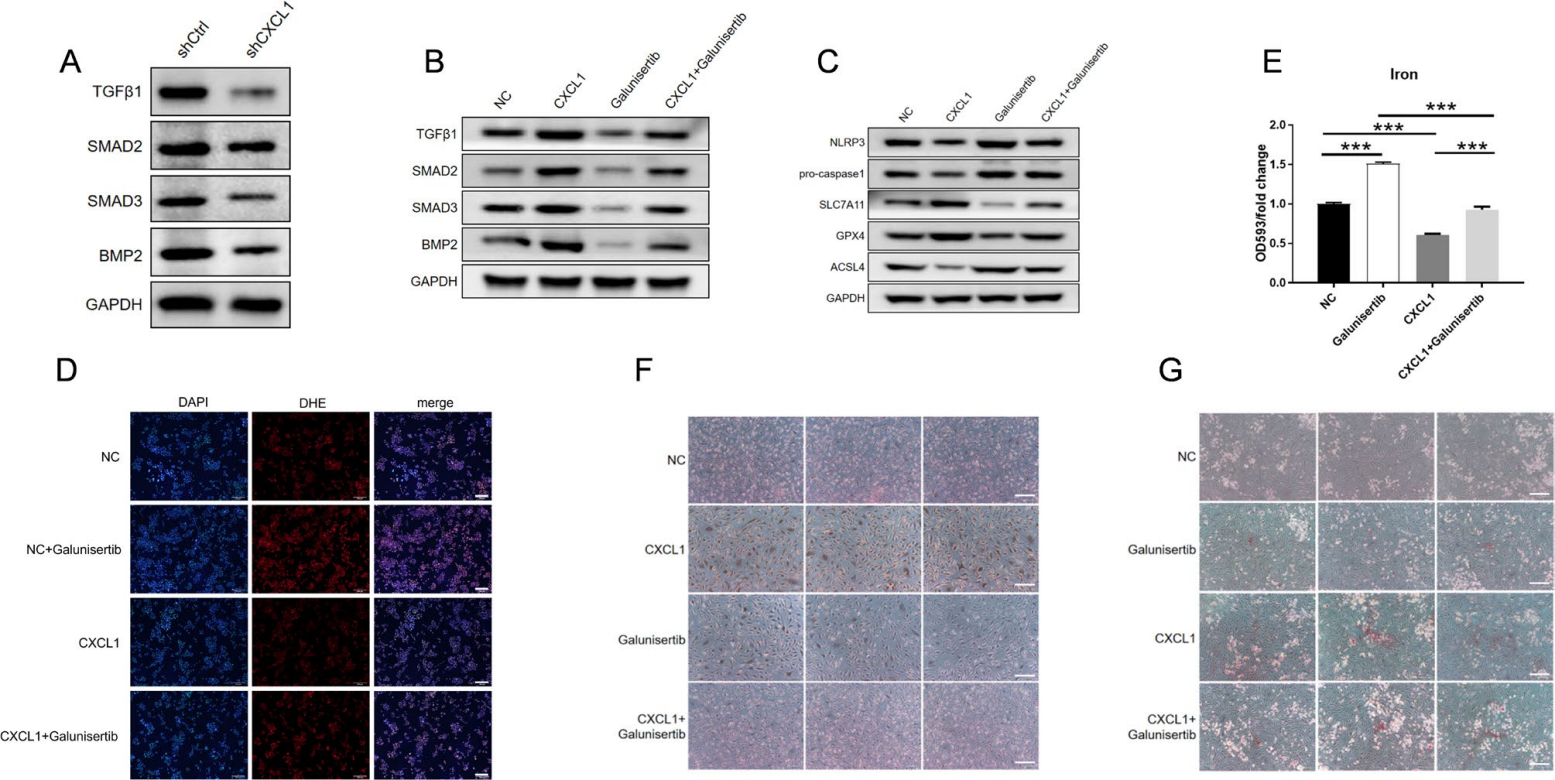
CXCL1 activates the TGF‐β1/Smad pathway to inhibit ferroptosis and promote osteoblast proliferation. Osteoblasts were assigned to the following groups: Control (shCtrl), CXCL1 knockdown (shCXCL1), untreated (NC), CXCL1 recombinant protein treatment (CXCL1), TGF‐β receptor kinase inhibitor treatment (Galunisertib), and CXCL1 recombinant protein with TGF‐β receptor kinase inhibitor treatment (CXCL1+Galunisertib). Western blot analysis (A, B) was conducted to measure the expression of TGF‐β1, Smad2, Smad3, and BMP2 proteins. Western blot (C) was used to detect ACSL4, SCL7A11, and GPX4 protein expression. ROS assay kit (D) was used to assess intracellular oxidative stress levels. Iron ion assay kit (E) was employed to determine intracellular iron content. ALP and Alizarin Red staining (F, G) were used to evaluate osteoblast differentiation capacity. Quantified values were mean ± standard deviation (SD, *n* = 5). Statistical significance was indicated by ***p < 0.01 compared to the “shCtrl” group. These experiments were repeated five times, consistently yielding similar results. Scale Bar = 200 μm.

Previous research indicates that TGF‐β signalling may heighten susceptibility to ferroptosis [17, 18]. Here, CXCL1 knockdown reduced the expression of TGFβ‐1 and downstream proteins Smad2, Smad3 and BMP2 (Figure 3A). Recombinant CXCL1 (10 ng/mL, 12 h) or the TGF‐β receptor kinase inhibitor Galunisertib (10 μM, 12 h) was used to validate pathway involvement. CXCL1 was shown to counteract Galunisertib's inhibition of the TGF‐β/Smad pathway (Figure 3B). Inhibition of TGF‐β/Smad signalling reduced SCL7A11 and GPX4, increased ACSL4 and elevated ROS and iron levels, leading to impaired differentiation, whereas recombinant CXCL1 mitigated these effects (Figure 3C–G). Thus, CXCL1 may regulate osteoblast differentiation by activating the TGF‐β/Smad pathway and inhibiting ferroptosis.

### 3.4 CXCL1 Promotes Autophagy Through the TGF‐β/Smad Pathway to Inhibit Ferroptosis

**FIGURE 4:**
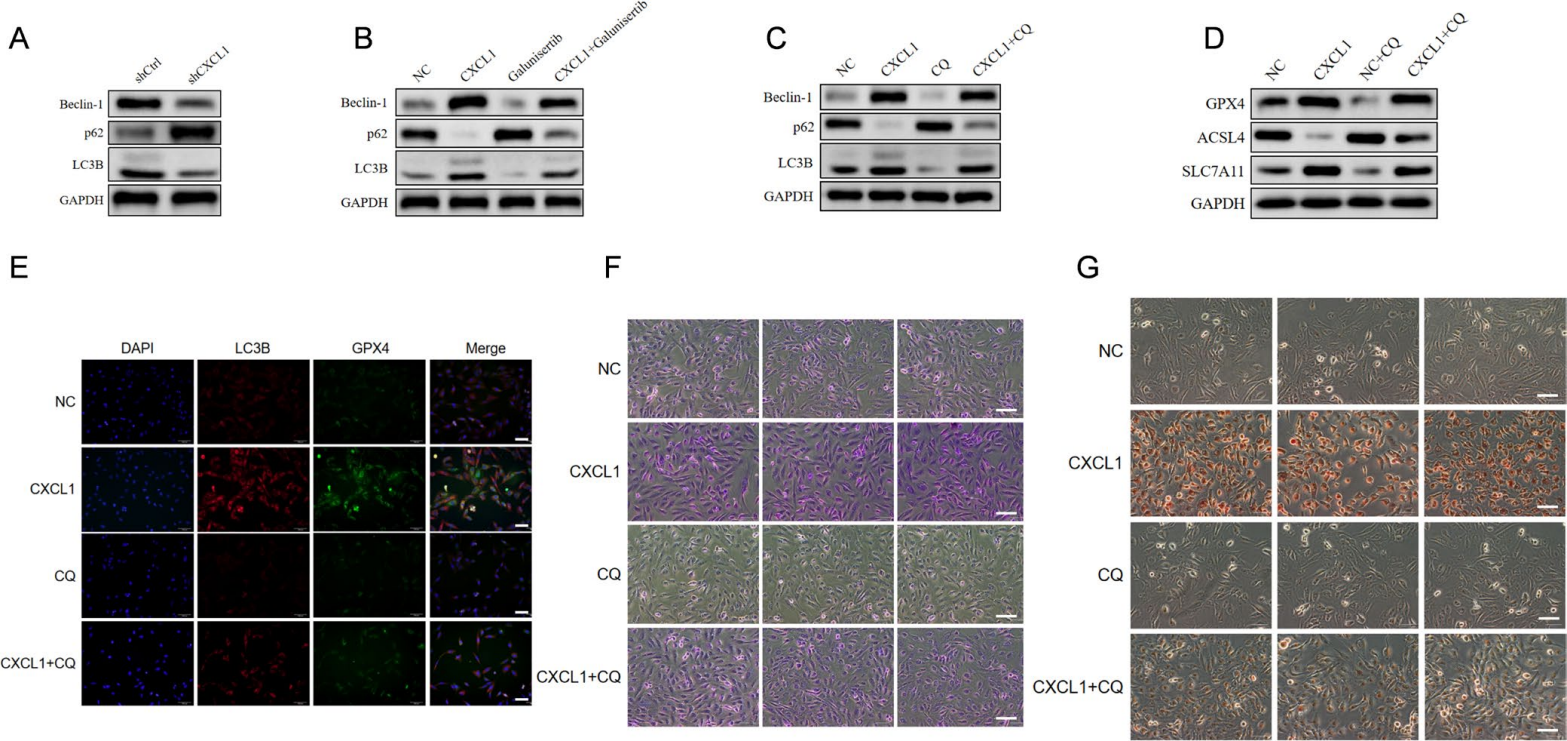
CXCL1 promotes osteoblast autophagy through the TGF‐β/Smad pathway, inhibiting ferroptosis and enhancing proliferation and differentiation. Osteoblasts were divided into the following groups: Control (shCtrl), CXCL1 knockdown (shCXCL1), untreated (NC), CXCL1 recombinant protein treatment (CXCL1), TGF‐β receptor kinase inhibitor treatment (Galunisertib), CXCL1 recombinant protein with TGF‐β receptor kinase inhibitor treatment (CXCL1+Galunisertib), autophagy inhibitor CQ treatment (CQ), and CXCL1 recombinant protein with CQ treatment (CXCL1+CQ). Western blot analysis (A–C) was used to detect the expression of autophagy‐related proteins p62, LC3, and Beclin‐1. Western blot analysis (D) was conducted to measure ACSL4, SCL7A11, and GPX4 protein expression. Immunofluorescence (E) was used to detect LC3B and GPX4 expression in osteoblasts. ALP and Alizarin Red staining (F, G) were used to assess osteoblast differentiation capacity. Quantified values were mean ± standard deviation (SD, *n* = 5). Statistical significance was indicated by *p < 0.05 compared to the “shCtrl” group. These experiments were repeated five times, consistently yielding similar results. Scale Bar = 200 μm.

Autophagy is essential for regulating ferroptosis in osteoblasts [19, 20, 21]. CXCL1 knockdown decreased autophagy markers Beclin‐1 and LC3B while increasing p62 (Figure 4A). Recombinant CXCL1 (10 ng/mL, 12 h) enhanced autophagy in osteoblasts, and inhibition of TGF‐β/Smad signalling by Galunisertib (10 μM, 12 h) reduced autophagy, indicating CXCL1's role in this pathway (Figure 4B). Further, autophagy inhibition with Chloroquine (CQ, 100 μM, 12 h) promoted ferroptosis markers, partially reversed by CXCL1 (Figure 4C–E). Reduced autophagy impaired osteoblast differentiation, which was partially rescued by CXCL1 (Figure 4F,G). Overall, CXCL1 promotes autophagy through the TGF‐β/Smad pathway, inhibiting ferroptosis and supporting osteoblast differentiation.

## 4 Discussion

Our results demonstrate for the first time that CXCL1 plays a protective role in osteoblast biology by modulating cell fate via autophagy and ferroptosis, mediated through the TGF‐β/Smad signaling pathway. The reduction of osteoblast viability and differentiation following CXCL1 knockdown, along with increased iron and ROS accumulation, aligns with a ferroptotic phenotype, highlighting a previously underappreciated function of CXCL1 in regulating oxidative cell death in bone cells.

Ferroptosis, a unique form of cell death driven by iron‐dependent lipid peroxidation, is increasingly recognised as a factor in bone metabolism disorders [22]. Iron overload, which leads to oxidative stress, disrupts the delicate balance of bone formation and resorption by triggering osteoblast cell death and promoting osteoclast activity [23, 24]. In our study, CXCL1 knockdown in osteoblasts resulted in increased intracellular iron and ROS levels, accompanied by upregulation of ferroptosis‐associated proteins such as ACSL4 and downregulation of protective proteins SLC7A11 and GPX4. This suggests that CXCL1 is crucial for maintaining osteoblast viability and function by countering ferroptosis‐related pathways. Interestingly, the partial restoration of osteoblast proliferation and differentiation upon treatment with the ferroptosis inhibitor Fer‐1 highlights that ferroptosis is a key mechanism through which CXCL1 affects osteoblasts. However, further in vivo studies will be essential to determine whether modulating CXCL1 expression can directly impact bone health and prevent osteoporosis progression in physiological settings.

The TGF‐β superfamily, including signalling molecules like BMP‐2, plays a critical role in bone homeostasis by promoting osteoblast differentiation and regulating osteoclast function [25, 26]. Our study also demonstrated that CXCL1 activates BMP‐2, promoting osteoblast differentiation and proliferation. Moreover, several studies have observed TGF‐β1‐induced activation of the canonical Smad pathway in osteoclast precursors and mature osteoclasts. For instance, Gratchev et al. reported that TGF‐β1 (10 ng/mL) could induce activation of the Smad2/3 signalling pathway as early as 10 min after stimulation [27]. Similarly, research by Ota et al. revealed that the expression of Wnt10b in response to TGF‐β1 (2 ng/mL) is dependent on Smad2/3 activation in osteoclasts but is independent of other pathways such as Akt or MAPK [28]. Consistent with these findings, our study showed that CXCL1 also activates the Smad2/3 signalling pathway in osteoblasts.

In cancer research, TGF‐β signalling is recognised as a bifunctional regulator, exerting tumour‐suppressive effects during early tumour stages but promoting tumour progression in later stages [29]. This dual role is primarily mediated through Smad‐dependent or Smad‐independent pathways [30]. Notably, the transcriptional regulation of CXCL1 involves the TGF‐β inhibitory element (TIE) located at −1247 bp upstream of the transcription initiation site and a Smad‐binding element (SBE) at −560 bp [31]. TGF‐β has been shown to suppress CXCL1 expression [32]. In the context of tumour progression, CXCL1 promotes tumour cell migration, thereby contributing to metastasis [33]. In contrast, our findings indicate that in osteoblasts, CXCL1 activates the TGF‐β signalling pathway to inhibit apoptosis, suggesting a possible reciprocal relationship between CXCL1 and TGF‐β signalling in osteoblasts and tumour cells. These results highlight the context‐dependent functions of CXCL1–TGF‐β signalling interactions in different cellular environments. Further investigation is needed to clarify whether CXCL1 functions upstream of TGF‐β signalling or acts through an alternative mechanism converging on Smad activation. Notably, our study initially planned to investigate the role of apoptosis‐related markers such as NLRP3 and pro‐caspase‐1. However, these molecules did not show consistent expression changes, nor did they correlate with ferroptosis markers. Given the limited relevance to our core mechanistic axis, we excluded them from the final analysis.

Autophagy is critical for cellular homeostasis and survival, allowing cells to degrade damaged organelles and proteins under stress conditions [34]. In osteoblasts, autophagy supports mineralization, differentiation, and overall bone health [35, 36]. Our findings indicate that CXCL1 knockdown reduces autophagy‐related proteins such as Beclin‐1 and LC3B while increasing the accumulation of p62, a marker of impaired autophagic flux. The ability of recombinant CXCL1 to restore autophagy marker levels, even under conditions of TGF‐β inhibition, suggests that CXCL1 promotes osteoblast autophagy, possibly as a protective mechanism against ferroptosis. Thus, the CXCL1–TGF‐β/Smad axis not only drives osteoblast differentiation but also establishes a protective feedback loop linking autophagy and ferroptosis regulation.

The observed crosstalk between autophagy and ferroptosis in our study aligns with recent findings that autophagy can mitigate ferroptosis by degrading ferritin and reducing free iron availability [37, 38]. In our experiments, inhibition of autophagy with the CQ inhibitor led to elevated markers of ferroptosis, reduced osteoblast differentiation, and increased oxidative stress—all of which were mitigated by CXCL1 administration. This supports the hypothesis that CXCL1 plays a protective role in osteoblasts by activating autophagy to control iron metabolism and prevent ferroptosis. This complex interplay between autophagy and ferroptosis in osteoblasts suggests that CXCL1 may act as a central regulator of cellular survival, particularly under conditions of oxidative stress and high iron availability. The ability to harness CXCL1's effects on autophagy and ferroptosis could open up new therapeutic approaches to enhance osteoblast function and improve bone quality.

Our findings position CXCL1 as a promising target for osteoporosis treatment. By promoting osteoblast autophagy and inhibiting ferroptosis, CXCL1 may help maintain bone homeostasis and prevent bone degradation associated with osteoporosis. CXCL1‐targeted therapies could complement existing osteoporosis treatments, particularly for patients with elevated oxidative stress or iron levels, which exacerbate bone loss. However, further studies are required to determine the effectiveness and safety of CXCL1 modulation in vivo.

Future research should explore how CXCL1 modulation affects bone remodelling in animal models of osteoporosis, assessing parameters such as bone density, biomechanical strength and histological changes in bone tissue. Additionally, investigating the effects of CXCL1 in combination with other TGF‐β/Smad pathway modulators or autophagy inducers may uncover synergistic effects that further enhance osteoblast function. Ultimately, translating these findings into clinical settings will depend on a thorough understanding of CXCL1's role in bone biology and its potential side effects on other tissues where CXCL1 is active, such as in inflammatory pathways.

## 5 Conclusion

This study elucidates CXCL1's role in regulating osteoblast function by inhibiting ferroptosis through the TGF‐β/Smad pathway, thereby enhancing osteoblast differentiation and proliferation. CXCL1 also promotes autophagy, further inhibiting ferroptosis to maintain osteoblast function. These findings identify CXCL1 as a potential therapeutic target for osteoporosis treatment, suggesting new avenues for research into bone metabolic disorders. Further in vivo studies are warranted to evaluate the translational potential of CXCL1‐targeted therapies in clinical settings.

## Author Contributions

**Zhiqiang Zhou:** conceptualization (equal), supervision (equal), writing – original draft (equal), writing – review and editing (equal). **Han Wu:** data curation (equal), investigation (equal), methodology (equal). **Qiong Chen:** methodology (equal), resources (equal), software (equal). **Jiao Sun:** investigation (equal), validation (equal), visualization (equal). **Huilan Li:** data curation (equal), formal analysis (equal), investigation (equal), validation (equal). **Jun Hua:** data curation (equal), investigation (equal), project administration (equal). **Dong Liu:** conceptualization (equal), funding acquisition (equal), supervision (equal), writing – original draft (equal), writing – review and editing (equal). **Yixin Shen** conceived and designed the study, and modified the manuscript. **Zhiqi Gao** carried out the molecular biology studies, and drafted the manuscript. **Yi Wang** and **Zhiqi** **Gao** performed the statistical analysis.

## Ethics Statement

Experimental protocols of this study were approved by the Ethics Committee of the Second Affiliated Hospital of Soochow University.

## Conflicts of Interest

The authors declare no conflicts of interest.

## Supporting information


**Table S1:** jcmm70883‐sup‐0001‐TableS1.docx.

## Data Availability

All data generated or analysed during this study are included in this published article. Data available on request from the authors.
