# Supplementary material for: CXCL1 Promotes Osteoblast Autophagy and Inhibits Ferroptosis Through the Activation of the TGF‐β/Smad Signalling Pathway
Source: J Cell Mol Med. 2025 Oct 30;29(21):e70883. doi: 10.1111/jcmm.70883 (PMC12573473; doi:10.1111/jcmm.70883)
Supplement: Supplementary file 1 — Table S1: jcmm70883‐sup‐0001‐TableS1.docx. [file JCMM-29-e70883-s001.docx]

Table 1

| Primer name Forward Reverse |
| --- |
| CXCL1 ACCCAAACCGAAGTCATAGC GGGACACCCTTTAGCATCTT |
| GAPDH GCAAGTTCAACGGCACAG GCCAGTAGACTCCACGACAT |
